# Assessment of radioactive substance transfer and its ecological and health impacts on the Nasser Lake ecosystem

**DOI:** 10.1038/s41598-025-09342-y

**Published:** 2025-07-18

**Authors:** Khaled Ali, Abd El-Basset Abbady, Ahmed Abu-Taleb, Shaban Harb

**Affiliations:** Physics Department, Faculty of Science, South Valley University, Qena, Egypt

**Keywords:** Nasser Lake, Radioactive contaminants, NORM/TENORM, Aquatic food web, Environmental impact, Radionuclide transfer, Health risk assessment, Environmental sciences, Natural hazards, Risk factors

## Abstract

This study investigates the distribution, transfer, and potential ecological risks of naturally occurring radioactive materials, including radon-222 (^222^Rn), radium-226 (^226^Ra), thorium-232 (^232^Th), and potassium-40 (^40^K), in the aquatic ecosystem of Nasser Lake, Egypt. As Egypt’s largest freshwater reservoir and a critical source of drinking water and fish, Nasser Lake plays a key role in environmental stability and public health. A total of 40 environmental samples—sediment, water, aquatic plants, and fish—were collected from 10 strategically selected sites around the lake. Gamma spectroscopy using sodium iodide activated with thallium [NaI(Tl)] detectors and AlphaGUARD radon monitoring systems was employed to measure radionuclide activity concentrations. Spatial distribution patterns were analyzed using Geographic Information System (GIS) techniques to identify zones of elevated radioactivity. The highest concentrations of ^226^Ra, ^232^Th and ^40^K were recorded in sediment samples near the High Dam, reaching 10.99 ± 0.42 Bq kg^−1^, 23.94 ± 1.91 Bq kg^−1^, and 277.38 ± 23.86 Bq kg^−1^, respectively. A strong positive correlation (Pearson’s r = 0.913) was observed between ^226^Ra and ^222^Rn exhalation rates, confirming that sediment accumulation significantly contributes to local radiological emissions. Bioaccumulation studies showed progressive uptake of radionuclides along the aquatic food chain, with fish exhibiting a bioaccumulation factor (BAF) of 0.74 for ^226^Ra. Estimated annual radiation doses from fish consumption reached up to 6.435 microsieverts per year (µSv y^−1^), remaining below international reference levels established by the World Health Organization (WHO). However, the combination of localized contamination near the High Dam and high fish consumption in nearby communities may present long-term radiological exposure risks. These findings highlight the importance of continuous monitoring of radioactive contaminants in sediment, water, and aquatic organisms in Nasser Lake. The study also provides a transferable framework for assessing the behavior of technologically enhanced naturally occurring radioactive materials (TENORM) in freshwater environments and supports the goals of the United Nations Sustainable Development Goals (SDGs) for clean water and good health.

## Introduction

Nasser Lake is one of the largest artificial lakes in the world. It was formed by the construction of the High Dam in Aswan, Egypt. The lake serves as a vital freshwater reservoir for biodiversity and surrounding communities. It supplies ample freshwater and supports a variety of aquatic plants, fish, and wildlife, benefiting nearby communities by providing resources for farming and fishing activities^1,2^. Runoff from phosphate fertilizers containing uranium and radium-226 (^226^Ra), along with industrial discharges from mining, have elevated levels of naturally occurring radioactive materials (NORMs) in the lake^3^. These activities have raised the concentrations of key radionuclides, such as ^226^Ra, thorium-232 (^232^Th), and potassium-40 (^40^K), above natural background levels, classifying the lake as a TENORM (technologically enhanced naturally occurring radioactive material) site ^4^^,^^5^^,^^6^. Radionuclides accumulate in sediments, plants, and fish, posing risks to ecosystems and human health^7^. Plants absorb radionuclides from water or through roots. Fish accumulate them directly from water or indirectly by eating contaminated organisms, enabling trophic transfer through the food web^8^^,^^9^^,^^10^^,^^11^. Furthermore, radon-222 gas (^222^Rn) emission from sediments is another source of radiation in the aquatic environment, affecting the lake’s water, plants, and fish. While previous studies focused on the distribution of natural radioactivity^7^^,^^12^^,^^13^^,^^14^^,^^15^, none have integrated ^222^Rn exhalation rates with trophic transfer in aquatic food webs, leaving key exposure pathways unquantified. This study addresses that gap by investigating the transfer and accumulation of radioactive elements in Nasser Lake’s ecosystem through a comprehensive analysis of their concentrations in water, sediments, aquatic plants, and fish. The general objective of this study is to understand and evaluate the impact of radioactive contamination in Nasser Lake, with particular focus on the risks associated with bioaccumulation in aquatic organisms. This study (1) quantifies ^226^Ra, ^232^Th, and ^40^K levels in water, sediments, aquatic plants, and fish using gamma spectroscopy; (2) assesses ^222^Rn exhalation from sediments; and (3) models radionuclide transfer through the aquatic food web to evaluate potential human health risks. Specific objectives include analyzing the concentrations of radioactive elements in water, sediments, aquatic plants, and fish; assessing the potential health impacts of these radioactive elements on aquatic organisms; and evaluating the risks of bioaccumulation in fish and plants, key components of the local food chain. Measurements of ^222^Rn emissions from sediments are included to provide a deeper understanding of the movement mechanisms of these contaminants. The study evaluates the health effects of radioactive elements on aquatic life and the risks of bioaccumulation in key food chain components like fish and plants. It also traces the transfer of radionuclides from sediments to plants and then to fish, directly linking sediment contamination to human health risks through dietary exposure—an aspect previously overlooked. Comprehensive analytical techniques, including gamma spectroscopy and ionization chambers, are used to measure radioactive element levels in various media and track their transport across the ecosystem. The findings are evaluated against international safety standards, particularly those provided by the World Health Organization (WHO)^5^, to evaluate the radiological risks associated with observed radioactivity levels. Our findings align with studies on TENORM-impacted lakes and provide a framework for monitoring radiological risks in man-made lakes globally, particularly those impacted by industrial activities, supporting sustainable development goals (SDGs) 3 (Good Health) and 6 (Clean Water)^16^. This study is essential for enhancing our understanding of Nasser Lake’s environmental conditions and highlighting the health risks posed by radioactive contamination^17^. This study underscores the urgency of adaptive management in TENORM-impacted aquatic ecosystems by linking sediment radioactivity to food web contamination. It supports the need for ongoing monitoring and management strategies to mitigate radioactive risks in the lake and protect both the environment and the health of local populations, especially in areas with significant sediment accumulation near the High Dam, where long-term exposure risks may develop.

## Experimental study

To capture the environmental variability of Nasser Lake, samples were collected from ten ecologically and geographically representative locations across the lake. Each location was selected to reflect diverse geological formations and aquatic ecosystem characteristics. To allow consistent cross-comparisons, ten samples of each type—water, aquatic plants, fish, and shore sediments— were collected per site. Coordinates using global positioning system (GPS) were recorded at each location to ensure consistency and reproducibility of sampling. Water samples were stored in 1-L Marinelli beakers suitable for gamma spectroscopy and transported under controlled conditions^7^^,^^18^. Fish were collected from local fishermen, cleaned, dried, and prepared using standardized procedures^19^. They weighed between 0.5 and 1 kg and were analyzed using specialist equipment. Aquatic plants were collected from the same sites, cleaned with deionized water, dried, ground into fine particles, and stored in Marinelli beakers. Shore sediments were collected 5–10 m from the water’s edge, dried at 105 °C, homogenized, and quartered before storage. To ensure homogeneity, sediments were ground into fine powders, dried at 105 °C for at least 24 h, homogenized, and then carefully combined^19^^,^^20^. The quartering method was used to obtain representative subsamples, which were subsequently stored in sealed Marinelli beakers. To ensure radioactive equilibrium between long-lived isotopes, such as ^226^Ra and ^232^Th, and their short-lived progeny, the samples were left to equilibrate for 28 days^19^. This procedure was designed to ensure accurate and reliable measurements^20^. Naturally occurring radioactive isotopes in four types of environmental samples—water, aquatic plants, fish, and shore sediments—were analyzed using gamma-ray spectrometry. The system consisted of a thallium-activated sodium iodide scintillation detector, NaI(Tl), with dimensions of 3 × 3 inches, coupled to a multichannel analyzer (Model 5510 Ortec Norland)^7^^,^^18^. The detector operated at a high voltage of 805 V DC, achieving an energy resolution of 7.5% at 662 keV and a peak efficiency of 2.3 × 10⁻^2^ at 1332 keV. To minimize background radiation, the detector was housed in a 50 cm thick cylindrical lead shield. All samples were measured in 1-L Marinelli beakers for 24 continuous hours to ensure statistically reliable data^7^. The system was calibrated using certified reference sources. Energy and efficiency calibrations were performed before each measurement session. Spectral analysis identified characteristic gamma photopeak’s corresponding to specific radionuclides. ^226^Ra activity was estimated through its decay products ^214^Pb and ^214^Bi at 295, 352, 609, 1120, and 1765 keV. ^232^Th activity was determined from its decay products ^228^Ac and ^208^Tl at 238, 911, and 2614 keV. ^40^K was identified by its gamma emission at 1460 keV. Activity concentrations (A) were calculated for each sample as either becquerels per liter (Bq L^−1^) for liquid samples or becquerels per kilogram (Bq kg^−1^) for solid samples, using the following equation^7^^,^^18^^,^^21^:

$$A=\frac{CPS}{E\times V\times P}$$

where *CPS* is the net count rate, *E* is the detector efficiency at the specific energy, *P* is the gamma-ray emission probability, and *V* is the sample volume or mass depending on sample type. The uncertainty in radiometric measurements was systematically assessed by considering all potential sources of error, following the international guidelines for uncertainty evaluation^22^. The statistical uncertainty (σ_*stat*_) was determined from the square root of the total net counts (√N) for each photopeak, where N represents the total counts under the peak after background subtraction. Systematic uncertainties (σ_sys_) were derived from multiple sources including P as reported in the International Atomic Energy Agency (IAEA) nuclear data libraries^23^, detector efficiency (E) obtained from calibration curves, and sample volume/mass (V) variations during preparation and measurement. The combined standard uncertainty (σ_*total*_) for each activity concentration was calculated using the root-sum-square method (RSS) as recommended in ISO 11,929–1:2019^24^:

$${\sigma }_{total}=\sqrt{{\left({\sigma }_{stat}\right)}^{2}+{\left({\sigma }_{sys}\right)}^{2}}$$

**Fig. 1:**
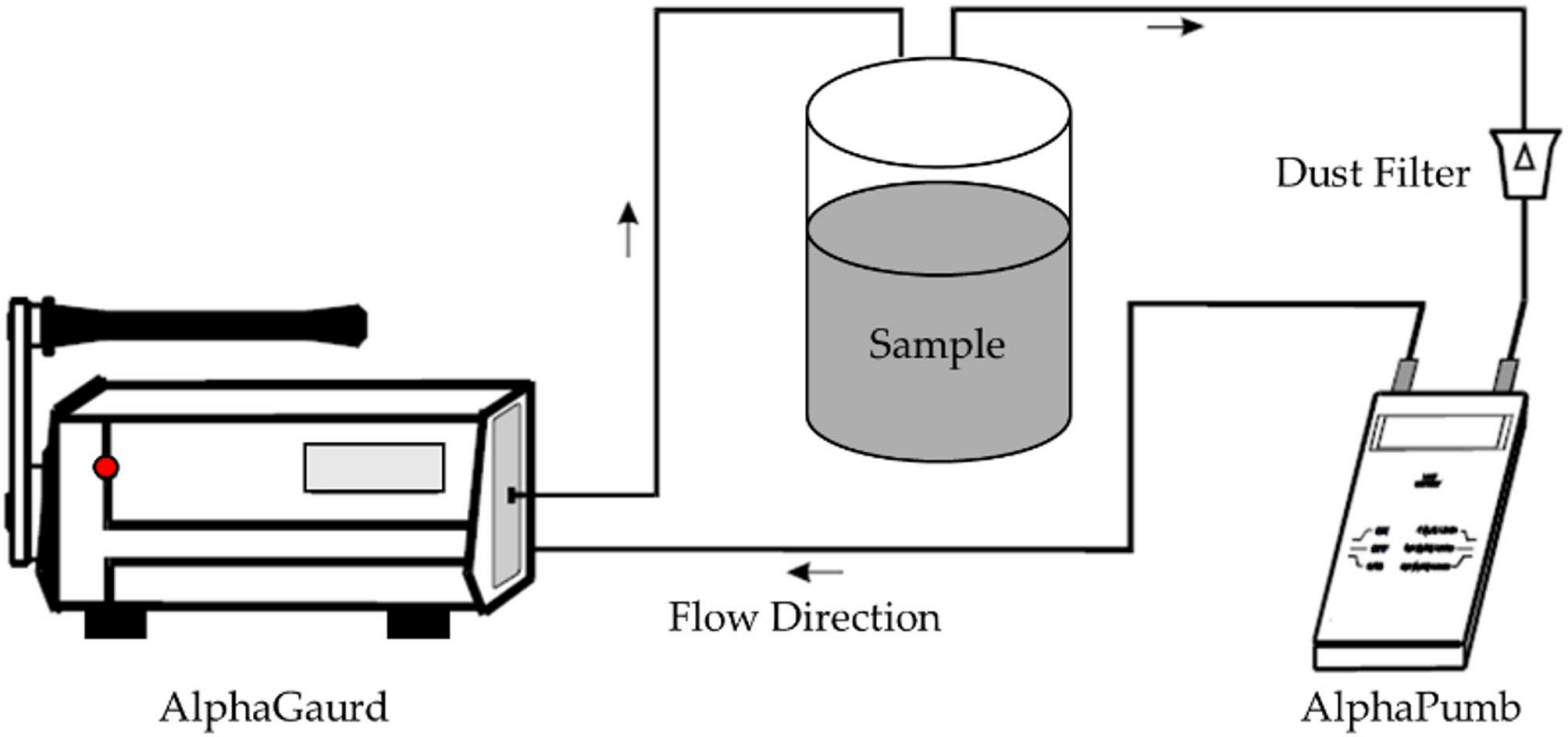
Application of AlphaPUMP in a closed gas cycle (emanation measurement in a closed vessel)

All results are presented as measured value ± expanded uncertainty at a 1 σ confidence level (k = 1). The relative standard deviation (%RSD) was additionally computed for each measurement to facilitate comparison between different samples, following standardized protocols for nuclear measurements^23^. To ensure measurement reliability, rigorous quality control measures were implemented in line with IAEA recommendations^23^. The system was calibrated prior to each measurement session using certified reference sources. Regular background measurements were performed and appropriately subtracted from all sample spectra. System stability was verified through periodic control measurements, and analytical accuracy was confirmed using certified reference materials. All reported uncertainties correspond to a 68% confidence level (k = 1) in accordance with international standards for measurement uncertainty evaluation^22^^,^^24^. Before measurement, solid samples (aquatic plants, fish, sediments) were dried, homogenized, sealed in Marinelli beakers, and stored for 28 days to achieve secular equilibrium between long-lived radionuclides (e.g., ^226^Ra, ^232^Th) and their short-lived progeny. This ensured accurate and reliable activity determinations^7^. ^222^Rn exhalation rate (^222^Rn_ex_) from shoreline sediment samples were measured using a combination of AquaKIT equipment and the AlphaGUARD PQ2000PRO ionization chamber^25^. The AquaKIT system includes key components such as a degassing vessel, the AlphaPUMP, a progeny filter, and the AlphaGUARD detector, all of which work together to measure ^222^Rn exhalation accurately. To begin the measurement process, the system was flushed with ambient air to adjust the baseline ^222^Rn levels, ensuring they matched the surrounding environmental concentrations. The background levels of the empty system were measured briefly before introducing the sediment samples^26^. Each sediment sample was placed into the degassing vessel connected to the AquaKIT system. The AlphaPUMP was then activated to circulate air through the vessel at a flow rate of 0.3 L min^−1^ for 10 min. This circulation process served to separate ^222^Rn from the sample. After the air pumping stopped, the AlphaGUARD detector continued measuring the ^222^Rn levels for an additional 20 min in flow mode. During this period, data were recorded every 10 min to monitor any fluctuations in the ^222^Rn concentration. To ensure the reliability of the results, the measurement process was repeated three times for each sample. Environmental factors such as temperature and humidity were carefully controlled throughout the experiment to minimize their impact on the measurements, ensuring accurate results^25^. All procedures followed internationally accepted standards, with proper calibration of instruments and repeated measurements to guarantee high-quality data and consistency^25^^,^^27^. Figure 1 illustrates the measurement system used in the experiments conducted with the AlphaGUARD detector. The figure shows the application of the AlphaPUMP in a closed gas cycle, which is essential for the emanation measurement in a sealed vessel. This system setup helps ensure precise measurements of ^222^Rn_ex_ from the sediment samples.

## Results and discussion

### Spatial distribution analysis of radioactive elements

**Fig. 2:**
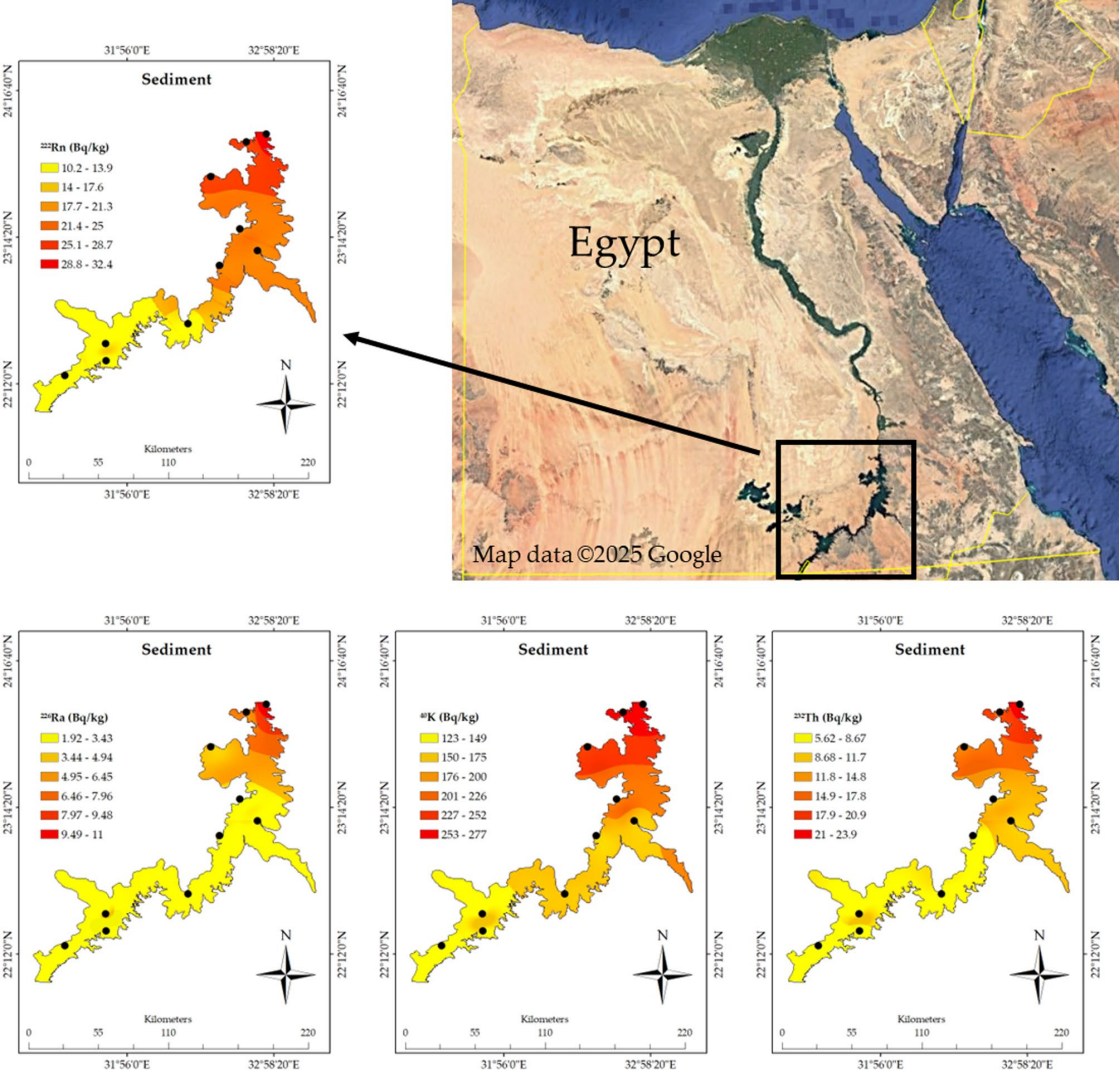
Spatial distribution of radioactive elements and their activity percentages (%) in sediments of Nasser Lake including (**a**) ^222^Rnex, (**b**) ^226^Ra, (**c**) ^232^Th, and (**d**) ^40^K. Egypt map created using Google Earth Pro Version 7.3 ^31^.

**Fig. 3:**
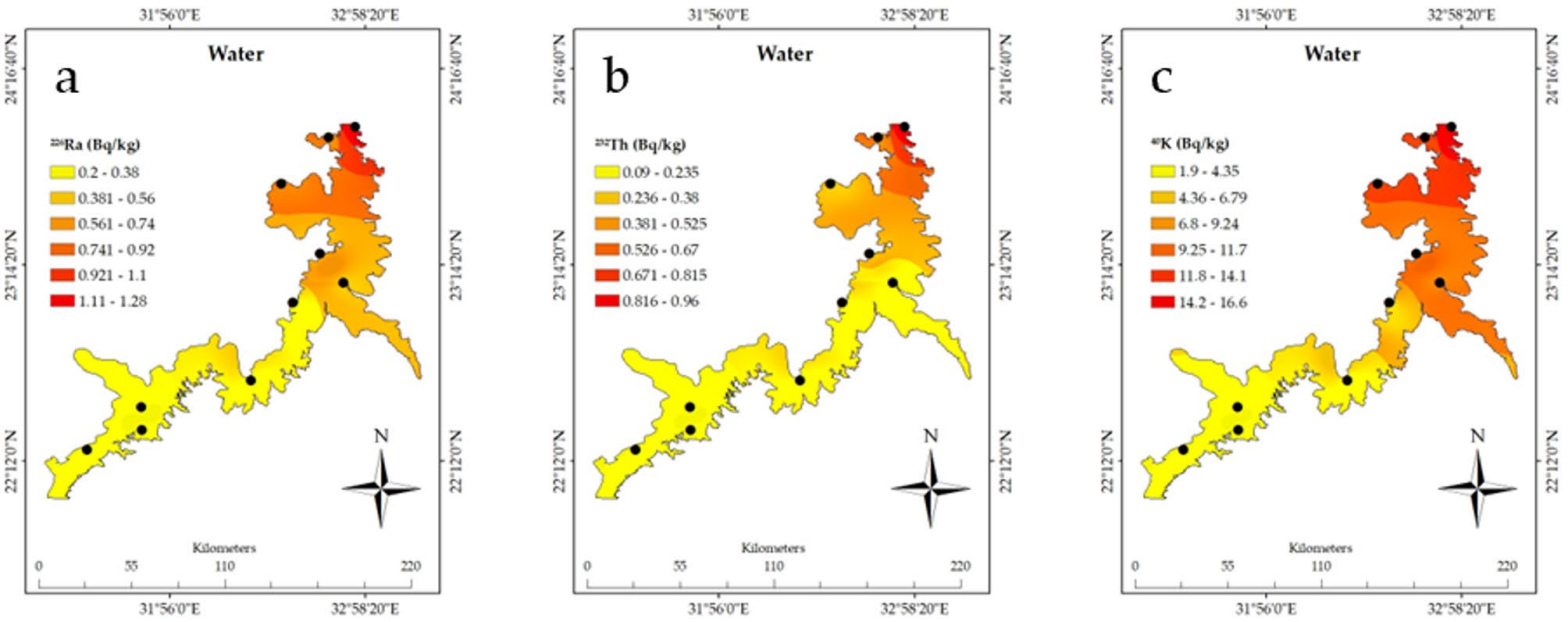
(**a** to **c**) Spatial distribution of radioactive elements in water of Nasser Lake including (**a**) ^226^Ra, (**b**) ^232^Th, and (**c**) ^40^K.

**Fig. 4:**
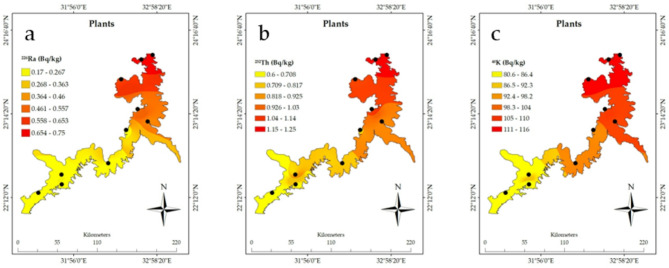
(**a** to **c**) Spatial distribution of radioactive elements in plants of Nasser Lake including (**a**) ^226^Ra, (**b**) ^232^Th, and (c) ^40^K.

**Fig. 5:**
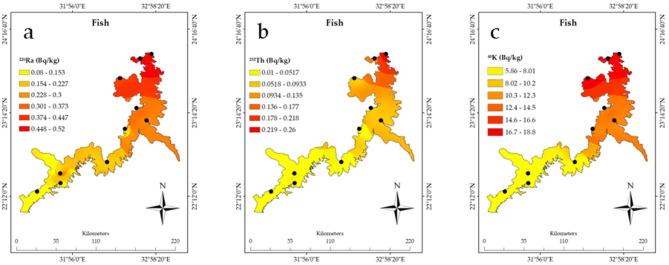
(**a** to **c**) Spatial distribution of radioactive elements in fish of Nasser Lake including (**a**) ^226^Ra, (**b**) ^232^Th, and (**c**) ^40^K.

The spatial distribution analysis using ArcGIS of the radioactive elements in Nasser Lake, based on the associated data in Table 1, clearly shows the significant influence of the High Dam on the distribution of these radionuclides. The measured radioactivity values from the sediment, water, plants, and fish samples were compared with the recommended values outlined by the WHO guidelines to assess the environmental and biological impacts of these radionuclides, with a specific focus on the sediment samples behind the High Dam, where increased NORM levels were observed^3^. Furthermore, the geological structure of the lake needs to be considered, as the characteristics of the rocks and sediments directly affect the accumulation and distribution of radionuclides. Figure 2a shows a noticeable spatial distribution of ^222^Rn_ex_ from the sediments, with the highest concentrations of ^222^Rn_ex_ measured near the High Dam at site 1 (23°58′11"N 32°55′18"E) measuring 32.36 ± 5.68 Bq m^-3^. In contrast, southern locations like site 8 (22°21′46.67"N 31°47′12.66"E) exhibited significantly lower levels of 10.16 ± 3.19 Bq m⁻^3^. These measured concentrations remain well below international safety standards, including the WHO recommended limit of 100 Bq m⁻^3^^28^ and the U.S. Environmental Protection Agency (EPA) action level of 148 Bq m⁻^3^^29^, confirming compliance with global guidelines for radon exposure. ^222^Rn concentrations tend to increase in areas near the High Dam due to sediment accumulation upstream. The dam prevents sediment flow into the Nile River, causing silt and heavy minerals to settle in this region. The Egypt map image shown in Fig. 2 was created using Google Earth Pro (Version 7.3)^30^. Studies show that these sediments, enriched by the geological characteristics of the surrounding area, contain notable concentrations of uranium. The Nile River carries heavy minerals, including uranium, from upstream regions, which accumulate in Nasser Lake due to the dam’s role in keeping sediment^1^. Additionally, the sedimentary rocks surrounding the lake, known for their uranium content, contribute to this enrichment^12^^,^^13^^,^^31^. While uranium concentrations in the area differ depending on location and sediment type, they are generally within natural distributions, showing the interaction of hydrological and geological processes. Fig. 2b depicts the distribution of ^226^Ra in sediments, with higher concentrations seen near the High Dam. Site 1 had a ^226^Ra concentration of 10.99 ± 0.42 Bq kg^−1^, while site 8 in the south had a significantly lower concentration of 1.92 ± 0.08 Bq kg^−1^. Such discrepancies show the considerable impact of the geological structure, where accumulations of sediment rich in radioactive elements near the High Dam result in increasing concentrations. Locations with uranium-rich rocks contribute to increased concentrations of these elements. As displayed in Fig. 2c, ^232^Th concentrations are significantly higher in locations closest to the High Dam. At site 1, the concentration was 23.94 ± 1.91 Bq kg^−1^, while site 8 recorded just 5.62 ± 0.28 Bq kg^−1^. The spatial distribution of ^40^K is similar to Fig. 2d, with higher concentrations at site 1 (277.4 ± 24 Bq kg^−1^) than at site 8, which recorded 123.27 ± 10.61 Bq kg^−1^. These findings confirm that the High Dam region is a hotspot for the accumulation of these radionuclides in sediments. When comparing the measured activity concentrations of ^226^Ra, ^232^Th, and ^40^K in sediments, particularly at Site 1 near the High Dam (10.99 ± 0.42 Bq kg^−1^ for ^226^Ra, 23.94 ± 1.91 Bq kg^−1^ for ^232^Th, and 277.38 ± 23.86 Bq kg^−1^ for ^40^K), with recommended values in international guidelines, it is important to note that the WHO does not specify strict reference values for radionuclides in sediments. However, typical background values for uncontaminated soil reported by the IAEA^32^ and UNSCEAR^33^ range between 17 and 60 Bq kg^−1^ for ^226^Ra, 7–50 Bq kg^−1^ for ^232^Th, and 100–700 Bq kg^−1^ for ^40^K. The levels observed in this study fall within or slightly below these global background ranges, indicating no regulatory exceedance but confirming the enhanced accumulation of radionuclides due to sedimentation processes near the High Dam. This context provides clearer evidence of localized elevation in natural radioactivity levels and supports the classification of the area as a TENORM-impacted site, reinforcing the need for periodic monitoring. Moving on to the water samples and the spatial distribution of radioactive elements presented in Figs. 3a–3c, the influence of the High Dam on radioactive element concentrations in the water is clear. For instance, Fig. 3a shows that site 1 had the highest concentrations of ^226^Ra in the water (1.28 ± 0.06 Bq L^−1^) compared to other sites. Figure 3b of the ^232^Th spatial distribution in water and Fig. 3c of the ^40^K spatial distribution in water show a parallel pattern, with concentrations considerably higher near the High Dam. These phenomena can be explained by the leaching of radionuclides from sediments into the water induced by the lake’s currents. In terms of plants, as shown in the spatial distribution in Figs. 4a–c, radioactive substance accumulation in plants is strongly correlated with geographic location. The concentrations are higher near the High Dam. For example, site 1 recorded 0.75 ± 0.04 Bq kg^−1^ of ^226^Ra; however, other sites showed lower concentrations. It shows that plants absorb radioactive elements more readily in places with higher concentrations in sediments and water. In the case of fish (Figs. 5a–c), radioactive substance concentrations are also higher nearthe High Dam. The fish at site 1 had the highest ^226^Ra content (0.52 ± 0.03 Bq kg^−1^), followed by ^232^Th (0.26 ± 0.02 Bq kg^−1^) and ^40^K (18.8 ± 1.6 Bq kg^−1^). These findings confirm the hypothesis that radioactive elements are transferred via the food chain, with radionuclides accumulating in fish due to their presence in water, plants, and sediments, highlighting the importance of understanding the radioactive pathway in the aquatic food web. Given the geological impacts of the High Dam, it is obvious that Nasser Lake’s geological structure has a major effect on radionuclide distribution, which is particularly evident in the food web chain where radioactive elements are transferred between aquatic organisms. The region’s sedimentary rocks, rich in uranium and other radioactive elements, together with the movement of water induced by the High Dam, play a key role in the accumulation of these elements in water, plants, and fish. As a result, the distribution of radioactive elements is not uniform, with concentrations higher near the High Dam and progressively decreasing as distance from it increases, which also impacts the transfer of radionuclides through the aquatic food web.

**Table 1:** ^222^Rnex (Bq m^-3^) from sediment and radioactive element concentrations (Bq kg^−1^) in sediment, plants, and fish, and (Bq L^−1^) in water samples.

Sites	Coordinates	Sediment				Water			Plants			Fish		
		^222^Rn_ex_	^226^Ra	^232^Th	^40^ K	^226^Ra	^232^Th	^40^ K	^226^Ra	^232^Th	^40^ K	^226^Ra	^232^Th	^40^ K
1	23°58′11"N 32°55′18"E	32.36 ± 5.68	10.99 ± 0.42	23.94 ± 1.91	277.38 ± 23.86	1.28 ± 0.06	0.96 ± 0.06	16.57 ± 1.43	0.75 ± 0.04	1.25 ± 0.08	115.84 ± 9.96	0.52 ± 0.03	0.26 ± 0.02	18.77 ± 1.61
2	23°08′37"N 32°51′34"E	22.32 ± 4.72	4.42 ± 0.18	12.26 ± 0.61	195.05 ± 16.78	0.62 ± 0.03	0.36 ± 0.02	10.30 ± 0.89	0.47 ± 0.02	0.98 ± 0.09	104.91 ± 9.02	0.34 ± 0.02	0.10 ± 0.01	13.08 ± 1.12
3	23°54′48"N 32°46′41"E	25.29 ± 5.03	7.02 ± 0.27	17.67 ± 0.91	257.01 ± 22.11	0.84 ± 0.04	0.54 ± 0.03	13.28 ± 1.14	0.69 ± 0.03	1.21 ± 0.07	114.54 ± 9.85	0.51 ± 0.01	0.17 ± 0.01	18.41 ± 1.58
4	23°40′08"N 32°31′36"E	25.16 ± 5.02	5.45 ± 0.21	16.77 ± 0.84	245.59 ± 21.13	0.77 ± 0.04	0.46 ± 0.03	11.88 ± 1.02	0.64 ± 0.03	1.07 ± 0.06	114.33 ± 9.83	0.43 ± 0.02	0.13 ± 0.01	17.92 ± 1.54
5	23°17′53"N 32°44′04"E	23.25 ± 4.82	4.58 ± 0.18	12.93 ± 0.65	209.95 ± 18.06	0.72 ± 0.04	0.39 ± 0.02	11.32 ± 0.97	0.53 ± 0.03	1.06 ± 0.07	108.29 ± 9.31	0.36 ± 0.02	0.10 ± 0.01	13.56 ± 1.17
6	22°21′46"N 31°47′12"E	15.55 ± 3.94	2.46 ± 0.1	8.23 ± 0.41	153.59 ± 11.18	0.26 ± 0.01	0.15 ± 0.01	2.58 ± 0.22	0.22 ± 0.01	0.70 ± 0.12	83.96 ± 6.11	0.18 ± 0.01	0.02 ± 0.00	6.19 ± 0.53
7	22°37′34"N 32°21′55"E	17.01 ± 4.12	4.14 ± 0.16	9.99 ± 0.5	184.03 ± 15.83	0.41 ± 0.02	0.25 ± 0.02	4.94 ± 0.43	0.30 ± 0.02	0.91 ± 0.06	100.2 ± 8.62	0.28 ± 0.01	0.03 ± 0.00	9.19 ± 0.79
8	22°15′33"N 31°29′40"E	10.16 ± 3.19	1.92 ± 0.08	5.62 ± 0.28	123.27 ± 10.61	0.20 ± 0.01	0.09 ± 0.01	1.90 ± 0.16	0.17 ± 0.01	0.60 ± 0.03	80.57 ± 6.93	0.080 ± 0.001	0.01 ± 0.001	5.86 ± 0.5
9	23°02′16"N 32°35′18"E	21.92 ± 4.68	4.28 ± 0.17	10.71 ± 0.56	186.71 ± 16.06	0.47 ± 0.02	0.29 ± 0.02	7.53 ± 0.65	0.34 ± 0.02	0.92 ± 0.06	101.28 ± 8.71	0.29 ± 0.02	0.09 ± 0.01	12.9 ± 1.11
10	22°29′08"N 31°47′00"E	16.39 ± 4.05	3.59 ± 0.14	9.7 ± 0.49	174.9 ± 15.05	0.38 ± 0.02	0.20 ± 0.01	3.76 ± 0.32	0.25 ± 0.01	0.86 ± 0.06	91.92 ± 7.9	0.24 ± 0.01	0.03 ± 0.00	6.34 ± 0.55

### Analyzing the correlation between radionuclide concentrations in aquatic ecosystems

#### Correlation analysis

Based on the correlation analysis (Pearson’s Correlation Coefficient) findings between radioactive element concentrations across aquatic ecosystems, the study shows strong and statistically significant correlations in the different media, and it is important to compare these concentrations with the recommended levels outlined in the WHO guidelines to better understand their environmental implications, especially in sediments near the High Dam. Table 2 presents a correlation matrix that details the correlations between aquatic ecosystems for each radioactive element.

**Table 2:** Correlation matrix of radioactive elements across aquatic ecosystems.

	Sediment to water	Sediment to plant	Water to plants	Water to fish	Plants to fish
^226^Ra	0.965	0.874	0.953	0.929	0.968
^232^Th	0.981	0.928	0.886	0.979	0.903
^40^ K	0.949	0.952	0.947	0.963	0.957

**Fig. 6:**
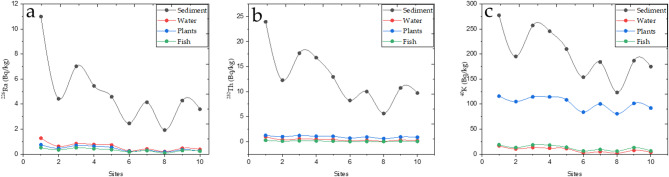
(**a** to **c**): Distribution of radioactive element concentrations in the aquatic ecosystems of Nasser Lake, including (**a**) ^226^Ra, (**b**) ^232^Th, and (**c**) ^40^K in sediments, water, plants, and fish, respectively.

The concentrations of ^226^Ra exhibited an intense correlation throughout aquatic ecosystems. The relationship between sediments and water showed the highest correlation coefficient value of 0.965, reflecting the balanced presence of ^226^Ra in both media. Meanwhile, the correlation between sediments and plants was 0.874, showing that ^226^Ra is transfered from sediments to plants, albeit to a lesser level than it moves to water. When comparing water and plants, the correlation coefficient was 0.953, showing a substantial relationship between ^226^Ra concentrations in both water and plants, implying that ^226^Ra in water has a clear influence on its accumulation in plants. Among the radioactive elements, ^232^Th had the highest correlation coefficient of 0.981, showing a significant correlation between ^232^Th concentrations in sediments and water. The correlation between water and plants was likewise robust, with a value of 0.886, showing a significant relationship between ^232^Th concentrations in water and plants. However, the correlation between plants and fish for ^232^Th was weaker compared to other element transfers, with a correlation coefficient of 0.903, showing that ^232^Th has a smaller effect on fish than its effect on plants. For ^40^K, the study found high relationships with other media. The correlation coefficient between sediments and water was 0.949, showing a strong correlation between ^40^K concentrations in sediment and water. The correlation between sediments and plants was 0.952, showing that sediments had a significant influence on ^40^K concentrations in plants. The plant-fish relationship was one of the strongest, with a correlation coefficient of 0.957, proving that ^40^K in plants can be successfully passed on to fish. Figures 6a–c show the distribution of radioactive concentrations in Nasser Lake’s aquatic ecosystems, with additional clarification needed on the specific areas within the lake that exhibit higher concentrations, which could be of significant concern for local ecological balance and biological health.

Based on these findings, it is clear that there are considerable correlations among radioactive elements across aquatic ecosystems, implying that the abundance of these elements in the environment is heavily influenced by their concentrations in other media. It is worth noting that sediments and water generally maintain equilibrium in terms of radioactive element concentrations, influencing ambient quantities of radioactive elements. Plants, on the other hand, play an important role in accumulating and transferring these components from other media, particularly ^40^K, to fish, and further investigation is needed to illustrate the full radioactive pathway, showing the transfer of radionuclides from sediments and water to aquatic organisms, including algae, fish, and other living species in the food web. These findings highlight the necessity of understanding the pathways through which these elements move within the ecosystem, as well as investigating their possible environmental effects on living organisms in Nasser Lake, including potential biological impacts on fish, plants, and other aquatic species due to accumulated radiation exposure.

#### Variance analysis

**Fig. 7:**
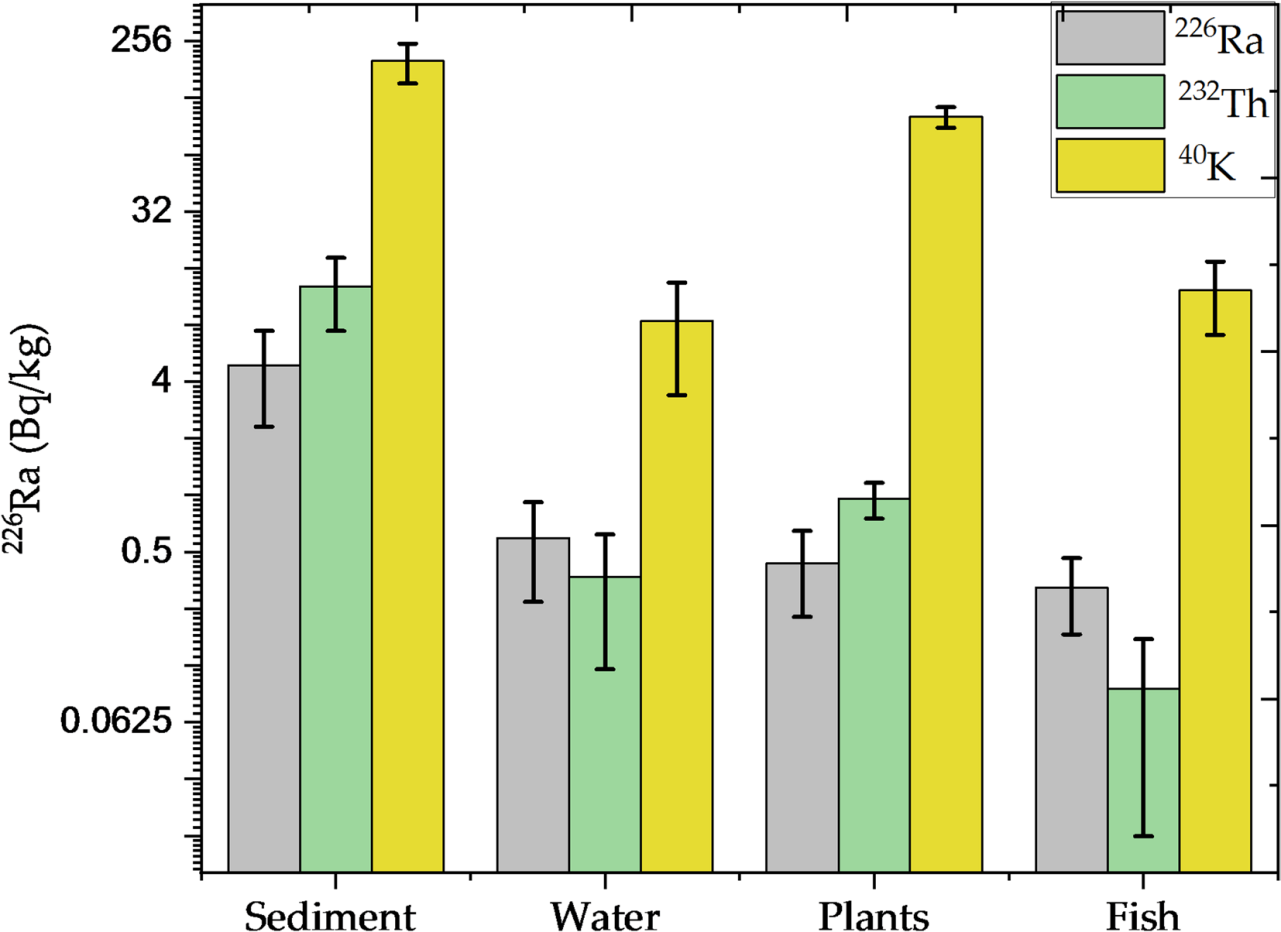
A grouped bar chart (log2 scale) displaying the average concentrations of radioactive elements in the aquatic ecosystems of Nasser Lake.

Table 3 shows the results of the variance analysis (ANOVA) for radioactive element concentrations in Nasser Lake’s aquatic ecosystems. The variance analysis for ^226^Ra across the aquatic ecosystems of Nasser Lake found extensive and significant variation. The *p*-value of 1.05 × 10^–9^ showed a significant difference in ^226^Ra concentrations across aquatic environments. Furthermore, the F-statistic value of 28.96, which is beyond the critical F-value of 2.866, provided added support for the finding of significant variations. The sum of squares (SS) values revealed that the between-group variation (147.8) contributed more to the overall variance than the within-group variation (61.23), supporting the conclusion that there are significant variations in ^226^Ra concentrations among aquatic ecosystems. The Tukey HSD analysis revealed significant variations between sediments and other aquatic ecosystems. The pairwise comparisons revealed significant variations between sediments and water, plants, and fish, with Q-statistics of 10.4, 10.79, and 11.06, respectively, and *p*-values < 0.01, indicating that sediments act as a primary source of ^226^Ra, with significantly higher concentrations than the other media. In contrast, no significant variations were found across water, plants, and fish, showing that the ^226^Ra concentrations in these mediums are identical. This highlights the importance of sediments as a reservoir for ^226^Ra in the Nasser Lake ecosystem and highlights the importance of comparing these concentrations with WHO recommended limits to assess potential health risks, especially in areas near the High Dam. The analysis for ^232^Th also revealed significant variations among the four media. The F-statistic value of 52.79, which exceeded the critical threshold of 2.87, and the *p*-value of 2.92 × 10^–13^, proved statistically significant variations between media. This reflects several reasons affecting variations in ^232^Th concentrations. The Tukey HSD test found significant variations between sediment and other aquatic ecosystems, with *p*-values < 0.01 for all comparisons (e.g., sediment vs. water, sediment vs. plant, sediment vs. fish). However, there were no significant variations between water, plants, and fish, as seen by the high *p*-values (> 0.01). These findings show that sediments function as a primary reservoir for ^232^Th, while ^232^Th transport between other aquatic ecosystems, such as water, plants, and fish, is less significant. Similarly, the analysis of ^40^K concentrations showed significant variations among media. The F-statistics of 132.44, along with a low *p*-value of 1.67 × 10^–19^, verified considerable fluctuation in ^40^K concentrations. The Tukey HSD test proved significant variations between sediments and water, plants, and fish (Q-statistics of 24.36, 12.56, and 23.88, respectively, with *p*-values < 0.01). It shows that ^40^K concentrations are significantly higher in sediments than in other media. Furthermore, there was significant variation between water and plants (Q-statistics of 11.80, *p* < 0.01), showing a large variance in ^40^K levels between these two mediums. There was no statistically significant variation between water and fish (Q-statistic of 0.48, *p* = 0.90) or between plants and fish (Q-statistic of 11.32, *p* < 0.01), suggesting that ^40^K levels in these media are more closely related. Lastly, the ANOVA and Tukey HSD tests proved that aquatic ecosystems have a considerable impact on radioactive element concentrations. Sediments appeared as the principal reservoir for these elements, with significant transfer occurs between sediments and water, plants, and fish, notably ^226^Ra and ^232^Th. However, at ^40^K, while sediments play an important role, the variations between water, plants, and fish were more subtle. These findings emphasize the importance of understanding how radioactive elements transfer through the ecosystem, particularly through the aquatic food web, and suggest the need to illustrate the potential pathways of radionuclide uptake by organisms such as fish and algae from sediments or water. Figure 7 displays the average radioactive element concentrations in Nasser Lake’s aquatic ecosystems.

**Table 3:** Results of ANOVA and Tukey HSD tests for radioactive element concentrations across the aquatic ecosystems in Nasser Lake.

Source of variation	SS	Degrees of freedom	Mean Squares	F-static	*P*-value	F crit	Treatments pair	Tukey HSD		
								Statistic	*p*-value	Inferfence
^*226*^*Ra*										
Between Groups	147.8	3	49.27	28.96	$1.05\times {10}^{-9}$	2.87	Sediment vs. Water	10.40	0.0010053	**p < 0.01
Within Groups	61.24	36	1.7				Sediment vs. Plant	10.79	0.0010053	**p < 0.01
Total	209.04	39					Sediment vs. Fish	11.06	0.0010053	**p < 0.01
							Water vs. Plant	0.39	0.8999947	Insignificant
							Water vs. Fish	0.66	0.8999947	Insignificant
							Plant vs. Fish	0.27	0.8999947	Insignificant
^*232*^*Th*										
Between Groups	1140.21	3	380.07	52.79	$2.92\times {10}^{13}$	2.87	Sediment vs. Water	14.63	0.0010053	**p < 0.01
Within Groups	259.19	36	7.2				Sediment vs. Plant	13.94	0.0010053	**p < 0.01
Total	1399.41	39					Sediment vs. Fish	14.95	0.0010053	**p < 0.01
							Water vs. Plant	0.69	0.8999947	Insignificant
							Water vs. Fish	0.32	0.8999947	Insignificant
							Plant vs. Fish	1.02	0.8832123	Insignificant
^*40*^* K*										
Between Groups	247,633.59	3	82,544.53	132.44	$1.67{\times 10}^{-19}$	2.87	Sediment vs. Water	24.37	0.0010053	**p < 0.01
Within Groups	22,436.9	36	623.25				Sediment vs. Plant	12.51	0.0010053	**p < 0.01
Total	270,070.49	39					Sediment vs. Fish	23.88	0.0010053	**p < 0.01
							Water vs. Plant	11.80	0.0010053	**p < 0.01
							Water vs. Fish	0.48	0.8999947	insignificant
							Plant vs. Fish	11.32	0.0010053	**p < 0.01

#### Multivariate analysis

**Fig. 8:**
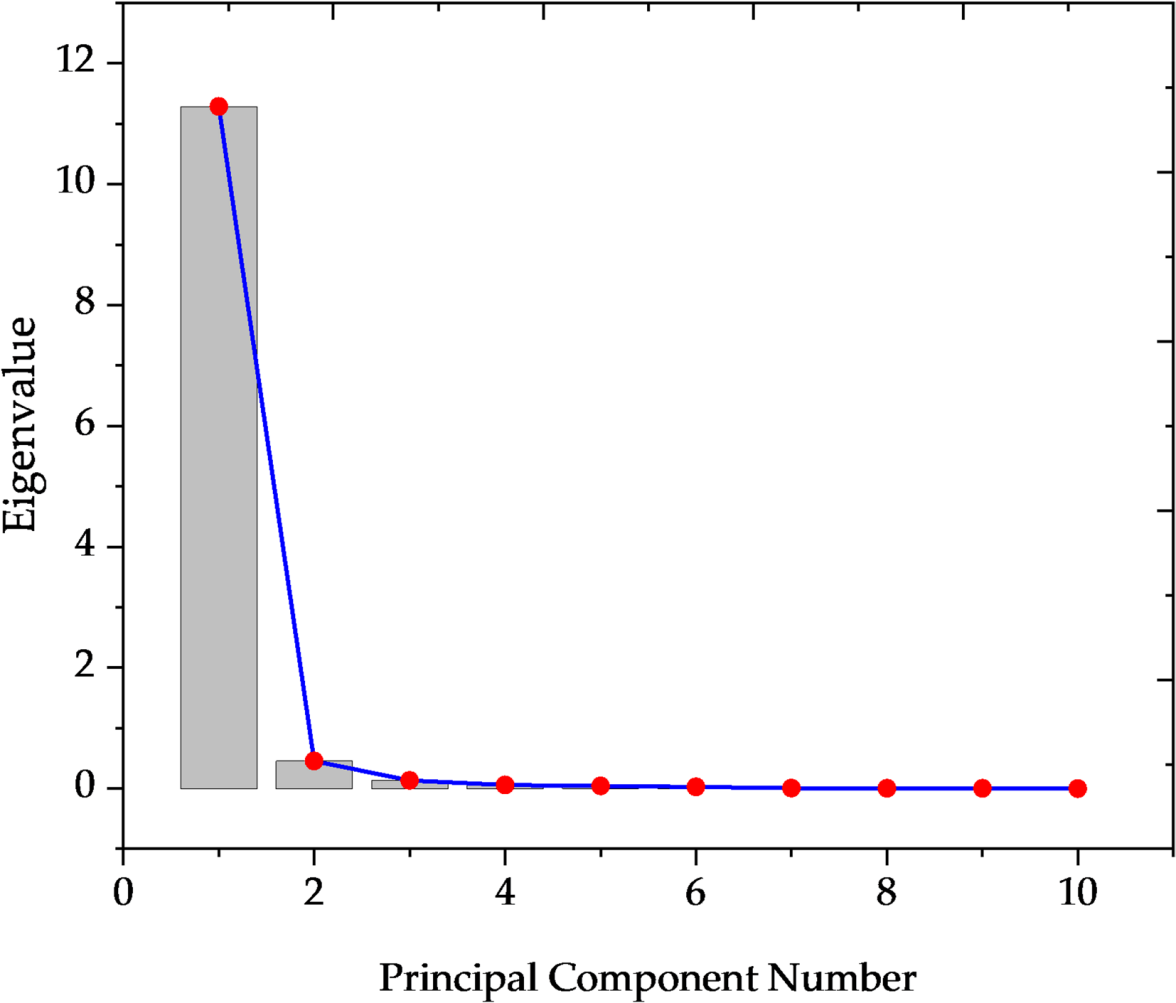
PCA plot of PCs vs. eigenvalue for radioactive elements in Nasser Lake.

Principal Component Analysis (PCA) is a statistical technique for reducing dimensionality and finding the underlying elements causing variance in multivariable data. In this study, PCA was used to examine the distribution of radioactive elements in Nasser Lake’s aquatic ecosystems. This analysis was an important component of the current study as it builds upon previous findings from ANOVA and Tukey tests, which helped reveal significant variations between the different locations in Nasser Lake. The PCA results, displayed in Table 4, clearly reveal that the first component (PC1) accounts for 94.04% of the entire variation in the dataset. This high proportion indicates that this component has a considerable effect on the data’s overall distribution. The PC1’s eigenvalue is 11.28, indicating its dominance in influencing variations across sample locations. This suggests that the majority of the variation in radioactive element concentrations can be attributed to the variables included in PC1, which most likely represent primary environmental factors, particularly the proximity to the High Dam and sediment accumulation behind it, which strongly influence the distribution of radioactive elements in the lake. In terms of the other components, the second component (PC2) explains just 3.77% of the variation, with the following components accounting for increasingly smaller quantities. This suggests that the secondary environmental variables represented by these components have a lower influence on the distribution of radioactive elements than PC1. The third through tenth components (PC3 to PC10) contribute truly little to explaining the variance, further emphasizing the dominance of PC1 in the analysis, Fig. 8. When correlating these results with the outcomes from ANOVA and Tukey tests, the PCA findings are further validated. The ANOVA and Tukey tests reveal significant variations in element concentrations across Nasser Lake locations, which are consistent with the PCA results, implying that large-scale environmental factors cause the majority of variance in radioactive element distribution. The significant proportion of variance explained by PC1 lends credence to the idea that variables such as proximity to the High Dam and the accumulation of sediment play an important role in determining the spatial distribution of these radioactive elements. The PCA findings provide valuable insight into the distribution of radioactive elements in Nasser Lake. They emphasize that the key environmental variables interact in a complicated way to influence the distribution of such elements. This analysis contributes to a better understanding of how environmental factors, particularly those related to the High Dam and the accumulation of sediments, affect the mobility and concentrations of radioactive elements in the lake ecosystem.

**Table 4:** PCA eigenvalues and variance explanation for radioactive elements in Nasser Lake.

PCs	Eigenvalue	Percentage of Variance	Cumulative
1	11.28426	0.9404	0.9404
2	0.45259	0.0377	0.9781
3	0.13171	0.0110	0.9890
4	0.05730	0.0048	0.9938
5	0.04037	0.0034	0.9972
6	0.02408	0.0020	0.9992
7	0.00572	0.0005	0.9997
8	0.00326	0.0003	0.9999
9	0.000701	0.0001	1
10	0	0	1

### Analysis of radioactive element accumulation in living organisms

#### Comparative assessment of radioactive element accumulation

The study of radioactive element accumulation in aquatic organisms (plants and fish) serves as an essential tool for understanding how radioactive waste affects the environment and human health. Determining the concentrations of radioactive elements in living organisms from the same regions around Nasser Lake helps clarify how these elements transfer through the food chain. These assessments assist in analyzing possible environmental concerns and identifying regions where radioactive accumulations may occur, impacting wildlife and human health. The findings of the Shapiro–Wilk test indicate that all data on radioactive element concentrations in living organisms indicate that the data are normally distributed. For plants, the p-values for ^226^Ra (0.43), ^232^Th (0.92), and ^40^K (0.32) are more than 0.05, confirming the hypothesis of normal distribution. For fish, the *p*-values for ^226^Ra (0.95), ^232^Th (0.216), and ^40^K (0.13) are all more than 0.05, indicating that the data is normally distributed. These findings enable the application of statistical tests such as the T-test to compare concentrations across plants and fish across multiple sites, allowing for more precise comparisons of radioactive elements in various ecosystems. When employing the T-test to examine the accumulation of radioactive elements in living organisms, some significant variations in element concentrations arise. Table 1 shows that ^226^Ra concentrations ranged from 0.17 ± 0.01 to 0.75 ± 0.04 Bq kg^−1^ in plants and 0.080 ± 0.001 Bq kg^−1^ to 0.52 ± 0.03 Bq kg^−1^ in fish. Given these differences, it is important to determine if the gap is significant enough to influence environmental findings. Plant concentrations of ^232^Th ranged from 0.60 ± 0.03 to 1.25 ± 0.08 Bq kg^−1^, whereas fish concentrations varied from 0.010 ± 0.001 to 0.26 ± 0.02. Plants had ^40^K concentrations ranging from 80.57 ± 6.93 Bq kg^−1^ to 115.84 ± 9.96 Bq kg^−1^, whereas fish had values ranging from 5.86 ± 0.50 to 18.77 ± 1.61, indicating significant variations across living organisms. The T-test will assess if the differences in radioactive element accumulation between living organisms are statistically significant. This investigation will help us understand how radiation transfers through the food chain and allow us to make more accurate environmental recommendations about radioactive hazards in the areas under investigation. To investigate the accumulation of radioactive elements in living organisms, a statistical analysis utilizing the T-test was performed to compare the accumulation of radioactive elements in living organisms from different locations. The analysis findings are shown in Table 5, which contains mean values, standard deviations, t-values, and p-values that establish the significance of variations in accumulation between living organisms.

**Table 5:** T-test Analysis for comparative assessment of radioactive element accumulation in the living organisms.

Element	Media	Mean (Mean ± STD)	t-value	*p*-value	Significance
^226^Ra	Plants	0.44 ± 0.21	1.42	0.173	Insignificant
	Fish	0.32 ± 0.14			
^232^Th	Plants	0.96 ± 0.21	12.39	< 0.0001	Significant
	Fish	0.09 ± 0.08			
^40^ K	Plants	101.58 ± 12.64	20.72	< 0.0001	Significant
	Fish	12.22 ± 5.14			

The T-test findings for ^226^Ra revealed no statistically significant variation in element accumulation between plants and fish (*p*-value = 0.173). This suggests that the variation in ^226^Ra accumulation among living organisms is not statistically significant. The average ^226^Ra accumulation in plants was 0.44 ± 0.21 Bq kg^−1^, whereas in fish it was 0.32 ± 0.14 Bq kg^−1^. Despite the modest variation in averages, the lack of significant changes implies that ^226^Ra accumulation is similar in both organisms. The T-test findings revealed a significant variation in ^232^Th accumulation across living organisms (*p*-value < 0.0001 and *t*-value = 12.39). The average ^232^Th accumulation in plants was 0.96 ± 0.21 Bq kg^−1^, whereas in fish it was 0.09 ± 0.08 Bq kg^−1^. These findings show that plants accumulate far larger amounts of ^232^Th than fish, indicating a difference in the ability to absorb this element amongst aquatic organisms. The T-test findings for ^40^K revealed a statistically significant variation (*p*-value < 0.0001 and t-value = 20.72). The average ^40^K accumulation in plants was 101.58 ± 12.64 Bq kg^−1^, whereas in fish it was 12.22 ± 5.14 Bq kg^−1^. The substantial variation in means implies that ^40^K accumulates significantly more in plants than in fish, which might imply that plants are more successful at absorbing this element than fish. The T-test helped assess if the differences in radioactive element accumulation between living organisms were statistically significant. The findings are consistent with the study’s overall conclusions, which show that variations in radioactive element accumulation are impacted by the nature of the organisms and their environmental interactions. The significant differences in ^232^Th and ^40^K accumulation are essential for understanding how these elements traverse the food chain, emphasizing the variation in their bioaccumulation across different organisms. According to WHO guidelines for radionuclide concentrations in food and drinking water, the measured levels of ^226^Ra, ^232^Th, and ^40^K in both fish and aquatic plants remain below the international safety thresholds for human consumption^34^. However, the elevated values in sediment near the High Dam indicate a potential for bioaccumulation over time, which underscores the need for periodic monitoring and long-term risk assessment. The statistical analysis using the T-test assisted in clarifying the statistical variations between the living organisms in the researched locations and allowed for enhanced comprehension of radioactive element transmission via the food chain in the studied environment. The uptake pathway of radioactive elements in Nasser Lake follows a typical aquatic food web model. Radionuclides such as ^226^Ra and ^232^Th first accumulate in bottom sediments, especially near the High Dam. Aquatic plants absorb these radionuclides through root uptake and direct contact with contaminated water. Herbivorous and omnivorous fish species then ingest radionuclides by consuming these plants or feeding on smaller organisms that feed on algae. This trophic transfer highlights the ecological significance of each organism’s role in the radioactive pathway, with plants acting as primary absorbers and fish as secondary accumulators. This investigation helps to understand how radiation moves through the food chain and allows for accurate environmental recommendations on radioactive hazards in the investigated locations. The aquatic food web in Nasser Lake plays a central role in the transfer of radionuclides through different biological components of the ecosystem. The process begins with the accumulation of radionuclides such as ^226^Ra, ^232^Th, and ^40^K in bottom sediments, particularly near the High Dam where sedimentation is most intense. These sediments act as the primary reservoir of radioactive contaminants. From this point, the radionuclides enter the water column through leaching and resuspension processes, making them bioavailable to aquatic organisms. Algae and aquatic plants serve as the first biological recipients of these radionuclides. Algae absorb them directly from the water, while rooted aquatic plants take up radionuclides both through their roots from contaminated sediments and via surface absorption from the surrounding water. These primary producers accumulate radionuclides and form the base of the aquatic food chain. Fish, which represent higher trophic levels, are exposed to radionuclides in two main ways: directly from the water and indirectly through dietary intake. Fish consume algae and aquatic plants, as well as smaller organisms that have already accumulated radionuclides, thereby enabling the upward transfer of radioactive elements through the food web. This trophic transfer highlights the specific ecological functions of each organism in the movement of radionuclides from abiotic sources (sediments and water) to living biota, especially fish. Understanding this pathway is essential for assessing long-term exposure risks to aquatic species and the potential radiological impacts on human health through fish consumption^8^^,^^9^^,^^11^^,^^35^.

#### Bioaccumulation factor of radioactive elements in living organisms

The bioaccumulation factor (BAF) between living organisms in an environment is critical to understanding how radioactive elements move through the food chain. This ratio is calculated using the following equation^36^:

$$\text{BAF}=\frac{\text{Element concentrations in fish}}{\text{Element concentrations in plants}}$$

**Fig. 9:**
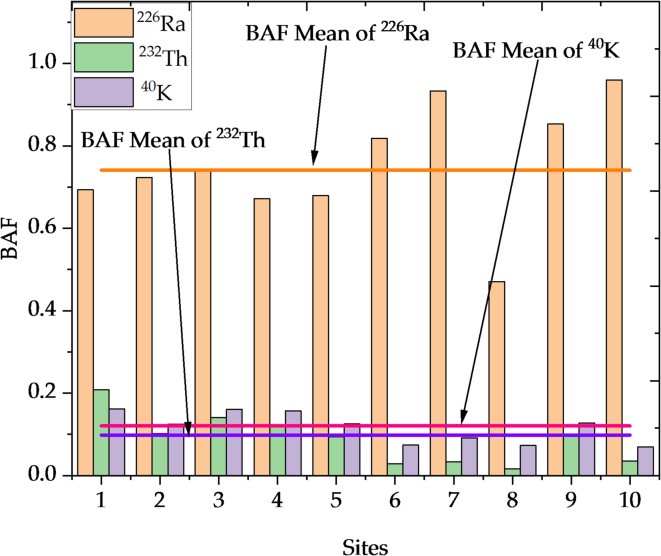
Bioaccumulation of radioactive elements in fish and plants across different sites in Nasser Lake.

This ratio indicates a fish’s ability to absorb and accumulate radioactive elements compared to plants. In addition to the numerical analysis given in Table 6, a graphical depiction of these findings is included in Fig. 9. The graphical depiction is made up of two overlapping plots: the first is a bar chart displaying the BAF at each location, and the second is a line plot overlay displaying the average BAF for each element across all locations.

**Table 6:** BAF Ratios of Radioactive Elements in Fish and Plants.

Sites	^226^Ra	^232^Th	^40^ K
1	0.69	0.208	0.162
2	0.72	0.102	0.125
3	0.74	0.14	0.161
4	0.67	0.12	0.157
5	0.68	0.094	0.125
6	0.82	0.028	0.0744
7	0.93	0.033	0.092
8	0.47	0.017	0.073
9	0.85	0.098	0.127
10	0.96	0.035	0.069
Mean	0.74	0.098	0.12

The accumulation of radionuclides in aquatic ecosystems follows a specific pathway. Radionuclides transfer from sediments to aquatic plants through root uptake or adsorption, and then to fish through ingestion. This bioaccumulation pathway highlights the role of each organism in transferring radioactive elements across the aquatic food web, especially in sediment-rich zones near the High Dam. The results of the bioaccumulation investigation, which assessed the concentrations of these elements in both fish and plants, emphasize organisms’ varying ability to accumulate radioactive contaminants. These findings were compared with the WHO guidelines for radionuclide limits in drinking water to evaluate potential ecological and human health risks^34^. For ^226^Ra, the BAF in fish varied significantly, ranging from 0.47 to 0.96, indicating that fish accumulate ^226^Ra more effectively than plants. This is particularly true for sedimentation areas near the High Dam, where sediment deposition and human activity increase the bioavailability of ^226^Ra. These locations have greater concentrations of ^226^Ra due to their strong interaction with fine sediment particles. The fish’s ability to absorb radioactive substances from water and sediment allows ^226^Ra to move up the food chain, magnifying its influence on the ecosystem. Fish accumulated increased amounts of ^226^Ra, which can have an impact on their health and survival, potentially altering trophic interactions and ultimately affecting aquatic biodiversity. The accumulation of ^232^Th in fish and plants was significantly lower, as evidenced by a BAF ranging from 0.016 to 0.208. ^232^Th’s significant affinity for sediments decreases its bioavailability and inhibits its accumulation in aquatic organisms. Despite its closeness to the High Dam, ^232^Th is primarily limited to sediments, reducing its influence on the environment. This restricted bioaccumulation decreases the immediate biological risk, but it does not remove the possibility of environmental disturbance. In contrast, ^40^K exhibited considerable bioaccumulation, with BAF values ranging from 0.068 to 0.16. Its critical involvement in cellular functions makes it more physiologically accessible across locations, although increased accumulation near the High Dam shows an interaction between biological processes and environmental conditions. While ^40^K is essential for aquatic organisms, its high concentrations near sedimentation sites demonstrate how human activity and natural processes combine to alter element distribution, potentially affecting local biodiversity.

The bioaccumulation of these elements in ecosystems has serious consequences for the environment. Fish’ health suffers when they acquire radioactive substances, potentially affecting their reproduction, growth, and immune response. These consequences extend up the food chain as carnivores that consume contaminated fish are also exposed to radiation, potentially leading to population declines and disrupting the aquatic ecosystem’s equilibrium. Furthermore, plants that absorb radioactive contaminants may experience stunted growth, development, and poor health, endangering food sources for herbivores and other organisms that rely on plants for survival. In terms of total biodiversity, radioactive contamination can lead to a decline in species diversity. Because certain species are unable to flourish in contaminated ecosystems, their populations decline, disrupting the ecological balance. The long-term repercussions might included a decline in the number of aquatic organisms, resulting in a less resilient environment. To summarize, the bioaccumulation of radioactive elements in aquatic organisms reflects a structured pathway of transfer through the food web, from sediment to plants and finally to fish. This process is influenced by sediment load, biological uptake mechanisms, and environmental factors. Understanding this pathway is essential for evaluating long-term ecological risks. Statistics indicate that locations with increased sediment accumulation, notably near the High Dam, have higher concentrations of radioactive elements in living ecosystems, which impacts the whole ecosystem. The findings highlight the necessity of continual monitoring and mitigating techniques for protecting biodiversity and aquatic ecosystem health.

### ^222^Rn Emission from sediments

The measurement of ^222^Rn emissions from sediments in Nasser Lake is an important step toward understanding the environmental impact of radiation in locations around lakes and dams. ^222^Rn is a radioactive gas produced by the accumulation of radioactive ^226^Ra in sediments^6^, and it has serious health and environmental consequences ^26^, particularly in locations where sediments accumulate near dams and areas with considerable human activity. The correlation between the concentrations of the radioactive elements and their effect on the surrounding environment can be better understood by comparing the measured radioactivity values with the WHO recommended limits for NORM and examining the association with the ^222^Rn emissions. This provides a clearer context for evaluating the potential risks of increased radiation levels, especially in areas near the High Dam. The correlation investigated between ^222^Rn emission in sediments and radioactive element concentrations revealed significant and unambiguous associations, demonstrating that these elements have a direct influence on ^222^Rn emission. The results demonstrated a significant relationship between ^226^Ra and ^222^Rn emission, with a Pearson correlation value of 0.913. This indicates that higher levels of ^226^Ra in sediments have a major impact on ^222^Rn emissions. Because ^226^Ra is the primary source of ^222^Rn in the environment, a direct relationship between ^226^Ra and ^222^Rn emission makes it logical. This finding shows that locations around the High Dam, which have a high level of ^226^Ra due to sediment accumulation, would have significant increases in ^222^Rn emission, as seen in Fig. 2a–d. The findings for ^232^Th revealed a 0.955 correlation with ^222^Rn emission, indicating that it has a considerable influence on ^222^Rn emission in sediments. Although ^232^Th does not directly decay into ^222^Rn, its presence in sediments increases its propensity to release ^222^Rn. ^232^Th aids in the transportation of ^222^Rn in the environment, especially in places with high levels of this element. The findings indicated a significant correlation (0.954) between ^40^K and ^222^Rn emission, which also suggests interactions that may influence the transfer of radionuclides through the aquatic food web. Although ^40^K is not a direct source of ^222^Rn, the correlation indicates interactions with other minerals in the environment that contain radioactive elements, which may lead to increased ^222^Rn emissions. ^40^K, an essential substance in rocks and minerals, helps to enhance radiation processes in these sediments. Based on these findings, it is possible to deduce that the concentrations of ^226^Ra and ^232^Th, the primary sources of this radioactive gas, have the greatest impact on ^222^Rn emission from sediments. Sediment locations with high levels of these elements, such as those near the High Dam, will see an increase in ^222^Rn emission rates. These findings underscore the necessity of investigating ^222^Rn emissions in these locations since ^222^Rn is a critical factor that may influence human health and the environment, demanding constant monitoring of these radioactive elements and the adoption of risk-mitigation strategies.

### Health risk analysis associated with radiation exposure through fish consumption

Nasser Lake, Egypt’s largest reservoir, is an important source of fish for the country as well as an important supply of fresh water for agriculture, drinking, and other human activities. While the presence of radioactive materials in the aquatic environment is a source of worry, that section concentrates on assessing the possible health hazards linked with their accumulation in Nasser Lake’s fish. The radiation doses humans may be exposed to by eating fish containing radioactive substances are determined. It is crucial to highlight that we did not address radiation doses in water since water goes through multiple processes of purification and desalination, which considerably reduces its radioactive content before it is utilized for drinking, irrigation, or other human activities. To calculate radiation doses from consuming fish, an equation was developed that connects the concentrations of each radioactive element in the fish to its associated dose conversion factor^18^^,^^37^. The equation applied is:

$${\text{Dose }}\left( {\mu Sv y^{-1} } \right) = \sum \left( \begin{gathered} {\text{Element }} {\text{concentrations }} in {\text{ fish}} \left( {{\text{Bq }} {\text{kg}}^{- 1} } \right) \hfill \\ \times {\text{Dose conversion }} {\text{factor}} \left( {\mu Sv {\text{ Bq}}^{-1}} \right) \hfill \\ \times {\text{Annual }} {\text{consumption}} {\text{ rate}} \left( {{\text{kg }} y^{-1} } \right) \hfill \\ \end{gathered} \right)$$

The calculations used three principal radioactive elements: ^226^Ra, ^232^Th, and ^40^K, with dose conversion factors of $2.8\times {10}^{-7}$ µSv Bq^−1^ for ^226^Ra, $2.3\times {10}^{-7}$ µSv Bq^−1^ for ^232^Th, and $6.2\times {10}^{-9}$ µSv Bq^−1^ for ^40^K^35^. According to government projections, an adult in Egypt consumes 20 kg of fish each year. Table 7 shows that radiation doses vary between locations based on their proximity to the High Dam. Statistics show that places closer to the High Dam get higher radiation doses, indicating a considerable deposit of radioactive materials in the sediments. Radiation doses fluctuated from 6.435 µSv y^−1^ near the High Dam to 1.221 µSv y^−1^ and 3.986 µSv y^−1^ at further out locations.

**Table 7:** Radiation doses (µSv y^−1^) for adults resulting from the accumulation of radioactive elements in fish from different locations in Nasser Lake.

Sites	^226^Ra	^232^Th	^40^ K	Total
1	2.912	1.196	2.327	6.435
2	1.904	0.460	1.622	3.986
3	2.856	0.782	2.283	5.921
4	2.408	0.598	2.222	5.228
5	2.016	0.460	1.681	4.157
6	1.008	0.092	0.768	1.868
7	1.568	0.138	1.140	2.846
8	0.448	0.046	0.727	1.221
9	1.624	0.414	1.600	3.638
10	1.344	0.138	0.786	2.268

**Fig. 10:**
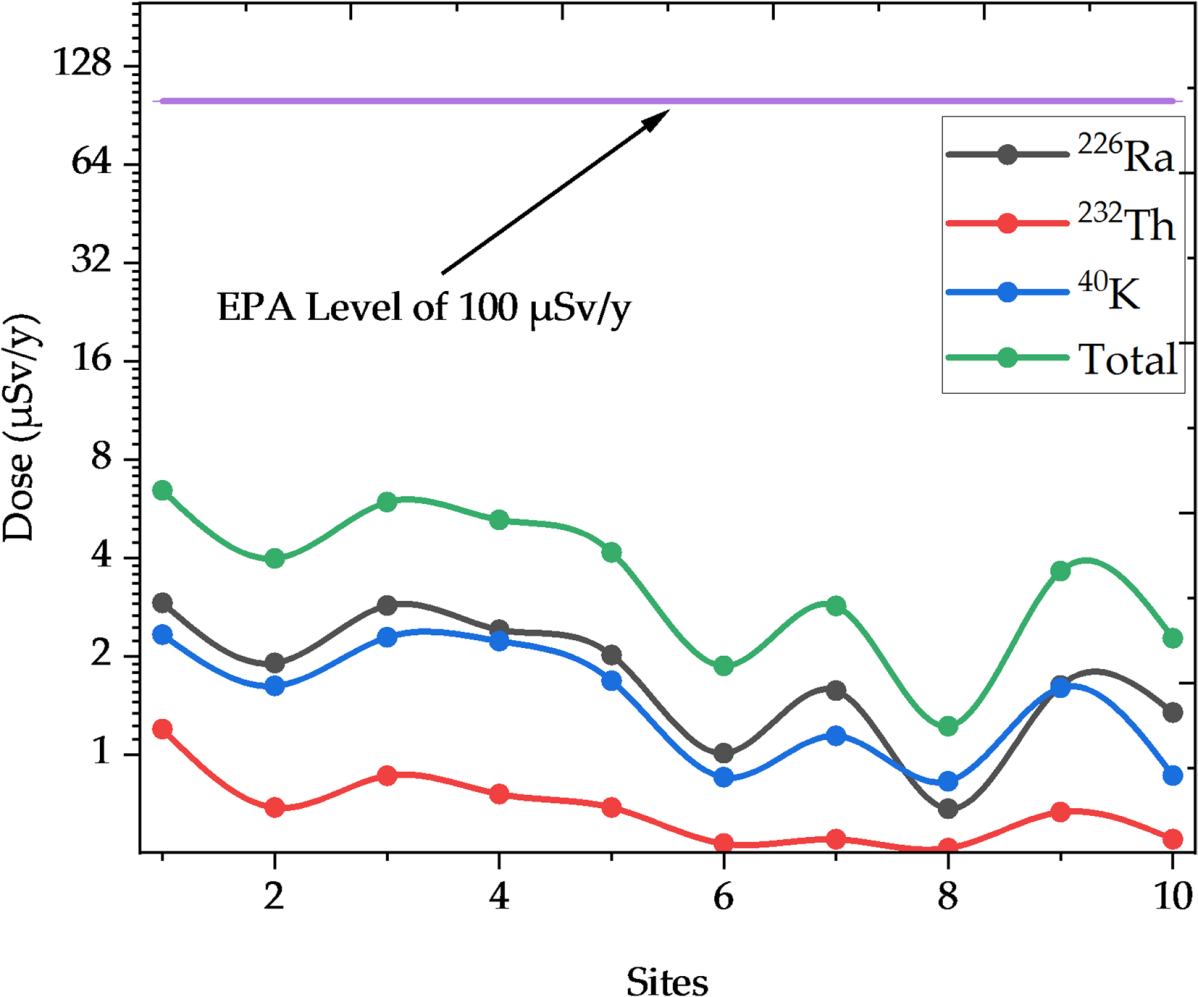
Spline connected chart (log2 scale) of radiation doses in Nasser Lake.

To further clarify these findings, a graphical representation in Fig. 10 is included that shows the distribution of radiation doses across the sample locations. The graph clearly shows an increase in doses in locations around the High Dam, which is consistent with earlier research suggesting a higher accumulation of radioactive elements in sediment areas near the High Dam. While the calculated radiation doses are low in comparison to the reference values set by the EPA, which states that the maximum annual radiation dose from food consumption should not exceed 100 µSv y^−1^^29^, it is essential to compare these levels with WHO-recommended reference values to assess the relative increase in NORM levels, especially in sediment samples behind the High Dam. Long-term consumption of fish may lead to radiation dose accumulation over time, indicating the potential for cumulative health effects. Thus, continuous surveillance of radiation doses is required to determine the possible health concerns among individuals who consume Nasser Lake fish on a regular basis.

This study sought to investigate the distribution and accumulation of radioactive elements in Nasser Lake, focusing on their potential transfer through the food web. It also aimed to assess the possible health concerns associated with human consumption of contaminated fish contaminated with these elements. The pathways of radionuclide uptake by aquatic organisms such as fish and algae from sediments, or through the food web, were analyzed to clarify the transfer pathways of these elements through the food web and accumulate in higher trophic levels. The study comprised a thorough examination of radioactive element concentrations in diverse aquatic ecosystems, including water, sediments, plants, and fish, at different locations around Nasser Lake. The use of statistical techniques was an important aspect of the study since it helped us understand the information being collected. Methods such as spatial distribution analysis, correlation analysis, variance analysis, and multivariate analysis shed light on the correlations between radioactive elements and their distribution in different ecosystems. The implementation of these statistical techniques allowed us a more accurate understanding of these elements’ activity across Nasser Lake, as well as their potential influence on aquatic life and human health. The study discovered that the majority of the radiation doses that people may be exposed to through fish intake remained within guidelines levels. However, locations near the High Dam have higher levels of contamination, indicating possible long-term hazards. This emphasizes the need for continued monitoring and more investigation to better understand the cumulative effects of radiation exposure over time. In closing, the study sheds light on the behavior of radioactive elements in Nasser Lake, emphasizing the importance of statistical analysis in drawing meaningful conclusions from complicated environmental data. While the findings provide valuable insight into the radiation doses from fish consumption, they underscore the significance of continuous monitoring, especially in areas near the High Dam. Additional research is necessary to fully assess the cumulative health risks associated with radiation exposure through the food web, ensuring a comprehensive risk assessment for the entire region.

## Conclusion

This study presents a comprehensive evaluation of the distribution and behavior of radioactive contaminants in Nasser Lake, Egypt, with a specific focus on their environmental and biological implications. The results revealed elevated concentrations of naturally occurring radionuclides, particularly radium-226 (^226^Ra) (up to 10.99 ± 0.42 Bq kg^−1^), thorium-232 (^232^Th) (up to 23.94 ± 1.91 Bq kg^−1^), and potassium-40 (^40^K) (up to 277.38 ± 23.86 Bq kg^−1^) in sediments, especially near the High Dam. These elevated levels are attributed to Technologically Enhanced Naturally Occurring Radioactive Materials (TENORM), primarily driven by phosphate fertilizer use, upstream sediment deposition, and anthropogenic influences. No artificial radionuclides, such as Cesium^−1^37, were detected, confirming that contamination originates from natural sources and technologically enhanced natural sources rather than nuclear fallout or industrial discharge. Strong correlations were observed between sediment radionuclide concentrations and radon-222 (^222^Rn) exhalation rates (r = 0.913 for ^226^Ra and r = 0.955 for ^232^Th), emphasizing the role of accumulated sediments as the principal source of elevated radioactivity in the lake’s ecosystem. Statistical analyses demonstrated significant differences in radionuclide concentrations among sediments, water, plants, and fish (ANOVA, *p* < 0.01), with sediments acting as the primary reservoir. Bioaccumulation was evident, particularly for ^226^Ra in fish (mean BAF = 0.74), followed by ^40^K (0.12) and ^232^Th (0.098), confirming the role of the aquatic food web in radionuclide transfer. Annual radiation doses from fish consumption ranged from 1.2 to 6.4 µSv year^−1^, remaining well below the international safety threshold of 100 µSv year^−1^ recommended by the U.S. EPA and WHO^29^^,^^5^. However, the elevated values observed near the High Dam suggest potential long-term ecological and human health risks, particularly for populations with high dependency on fish from the lake. When compared to other regional and international studies, the observed levels in Nasser Lake exceed background concentrations reported in upstream Nile water and other African dam reservoirs, highlighting the influence of the High Dam in altering radionuclide dynamics and sedimentation patterns. These findings underscore the need for sustained monitoring of radionuclide levels in sediments, water, and biota, regular assessment of public health risks for nearby communities, and stricter control of human activities contributing to TENORM accumulation. Future research should explore long-term ecological impacts, track radionuclide transfer through additional trophic levels, and confirm the absence of artificial radioactivity. Overall, this study contributes critical insight into the radiological status of Nasser Lake and supports the development of evidence-based strategies for environmental protection and health risk mitigation in the region.

## Data Availability

The datasets generated and/or analyzed during the current study are available from the corresponding author on reasonable request.
